# C2 domains as protein-protein interaction modules in the ciliary transition zone

**DOI:** 10.1186/2046-2530-4-S1-P65

**Published:** 2015-07-13

**Authors:** K Remans, M Bürger, I Vetter, A Wittinghofer

**Affiliations:** 1 Max Planck Institute for Molecular Physiology, Dortmund, Germany

## 

RPGRIP1 (RPGR interacting protein 1) is mutated in the severe eye disease Leber´s Congenital Amaurosis (LCA), while the structural homologue RPGRIP1L (RPGRIP1-like) is mutated in many different ciliopathies. Both are large multi-domain proteins predicted to interact with RPGR, the Retinitis Pigmentosa G-Protein Regulator. RPGR is mutated in X-linked Retinitis Pigmentosa and located in photoreceptors and in primary cilia. We solved the crystal structure between the RPGR-interacting domain (RID) of RPGRIP1 and RPGR and demonstrate that RPGRIP1L binds to RPGR in a similar mode. RPGRIP1 binding to RPGR affects the interaction with PDEð, the cargo shuttling factor for prenylated ciliary proteins. The RPGRIP1-RID is a type II C2 domain with a canonical ß-sandwich structure. However, it does not bind Ca^2+ ^and/or phospholipids and thus constitutes a new type of protein-protein interaction module. Judging from the large number of C2 domains present in nearly all ciliary transition zone proteins identified, the structure presented here seems to constitute a cilia-specific module present in multi-protein transition zone complexes.

